# Transitions in alcohol use over time: a survival analysis

**DOI:** 10.1186/s40359-020-00479-1

**Published:** 2020-11-03

**Authors:** Laura B. Koenig, Jon Randolph Haber, Theodore Jacob

**Affiliations:** 1 Winona State University, Winona, MN USA; 2 Veterans Affairs Palo Alto Health Care System, 795 Willow Road, MC 151J, Menlo Park, CA 94025 USA; 3 Department of Social Sciences, Southwest Minnesota State University, 1501 State Street, Marshall, MN 56258 USA

**Keywords:** Alcohol dependence, Heavy drinking, Relapse, Remission, Risk factors

## Abstract

**Background:**

The current study examined the predictors of the onset of alcohol use as well as predictors of remission and relapse, both from heavy drinking and from alcohol dependence. Similarities and differences in both clinical and psychosocial predictors across the transitions were examined.

**Methods:**

A sample of men from the Vietnam Era Twin Registry (N = 1769) completed an assessment of lifetime drinking history, which allowed age markers for starting and stopping different drinking patterns. The men also completed various assessments regarding personality, alcohol motives, and psychiatric diagnoses. Survival analyses were used to examine the predictors of the three transitions of onset, remission, and relapse for the phenotypes of heavy drinking and of alcohol dependence, censoring the individuals who had not yet experienced an event.

**Results:**

As expected, predictors of onset for drinking, heavy drinking, and alcohol dependence were largely consistent and included externalizing symptomology, nicotine dependence, and cotwin history of drinking as risk factors. Predictors of remission from heavy drinking, somewhat similarly to remission from alcohol dependence, included the risk factor of externalizing disorders but also, as predicted, included more risk and protective factors in the psychosocial realm that were not predictors of onset. Contrary to our prediction, relapse to heavy drinking and alcohol dependence were predicted largely by unique psychosocial risk and protective factors including social and coping motives.

**Conclusion:**

Current findings extend the findings of past research to remission and relapse in the later decades of life and have implications for treatment of alcohol use problems.

## Background

Researchers and clinicians agree that the course of alcohol dependence is better characterized as a widely varying process with varying consequences than as a uniform pattern of steadily declining health and functioning. Differences in the course of alcohol problem use and dependence have been identified and characterized via work on subtypes, etiology, treatment, and trajectories of alcohol use disorders [1–6]. For example, this research group has previously examined Vietnam Veterans with a lifetime diagnosis of alcohol dependence (AD) and found that only 8% of these men indicated their drinking had not changed significantly throughout their adult years [7, 8]. Instead, the drinking course of these veterans with alcohol dependence over a 25-year period involved an average of 4.2 drinking phases each lasting an average of 8.22 years, with both increases and decreases in severity and consequences. Differences in pattern, severity, and consequences appear to be the rule, not the exception. To capture this variation, a developmental view of alcohol dependence has gained increasing support [9], and the National Institute for Alcohol Abuse and Alcoholism (NIAAA) [10, 11] has emphasized examination of alcohol use disorders within a developmental, lifespan framework of intersecting biological and environmental vulnerabilities.

Although the prevalence of alcohol use disorders (AUD) is highest during adolescent and young adult years, treatment seeking for AUD is twice as likely to occur after age thirty [10], and more studies are needed that extend examination of alcohol use into the later decades of the lifespan. It is important to identify what proximal and distal influences account for remission of drinking (whether through treatment or not) and to identify the predictors of subsequent relapse to problem drinking if that occurs. Twin research had indicated that differences in remission phenotypes are largely due to environmental effects [12]. Remission from drinking has been associated with the recent onset of drinking problems; receiving greater social support; having friends who disapprove of drinking; experiencing more positive and fewer negative life events; gaining stronger coping behaviors; increases in meaning in life; experiencing less temptation to drink; having higher self-efficacy; and having fewer psychiatric or psychological disorders [13–15]. It has been found that premorbid social stability (especially stable employment history) predicts long term abstinence [16]. In contrast, relapse, not surprisingly, seems most common in individuals who had more severe alcohol use problems, for example, with higher rates of past alcohol use and more lifetime symptoms of alcohol dependence [17]. When considering more psychological risk factors, relapse following treatment has been found to be predicted by experiencing negative emotional and interpersonal problems [18] and reduced participation in outpatient treatment or Alcoholics Anonymous (AA) meetings [16]. Also, similarity has been identified between factors predicting the initiation of problem drinking and factors predicting relapse or patterns in use across time. These include having a family history of or genetic risk for alcohol dependence [1, 5, 19], lower family-of-origin socioeconomic status (SES) [20], certain personality and individual difference variables (e.g., being high in sensation seeking and disinhibition) [21, 22], perceived peer alcohol and drug use, difficulties with major role transitions (e.g., marriage, occupational changes) [23, 24], and having a comorbid psychiatric disorder (including both conduct problems and depression diagnoses) [25, 26]. Not only is there a wide cross-section of factors that appear to influence the course of alcohol use problems, similar factors may predict increasing alcohol-related difficulties at various stages, and other factors may predict decreasing difficulties in the overall course of alcohol dependence.

Moving beyond association studies of specific milestones in alcohol use are studies of the rate of progression from one stage of use to another. In such models, risk factors would be those influences that accelerate a person’s progression to the next stage and protective factors would be those that delay progression. Evidence shows that different psychiatric and psychosocial factors increase (or decrease) the rate of progression through a specific stage [27–29]. As would be expected, these factors are reasonably consistent with the factors identified in association studies focused on initiation, continued drinking, AD diagnosis, and varying trajectory severities.

A key limitation of association studies is that the predictors of any given milestone will necessarily include the predictors of all preceding milestones. That is, the predictors of AD will also include the predictors of initiation of drinking since initiation always precedes AD. Studies that model the rate of progression though a series of distinct sequential periods defined as beginning at one milestone and ending at another can isolate predictors of each transition. Survival analysis, which can examine the time it takes to experience a given event and what predicts a quicker or slower progression, is one such model. For example, in a study of young adults, Sartor et al. [29] used conditional survival analysis to examine risk factors associated with years to first drink and years from first drink to AD. Results indicated that although conduct disorder was a consistent predictor of both the progression to initiation and progression from initiation to AD, other factors predicted only one transition or the other (attention deficit hyperactivity disorder, parental AD, male gender, and parental divorce for initiation of drinking compared to nicotine dependence, cannabis abuse, and generalized anxiety disorder for subsequent progression to AD). This study was the first to differentiate between risk factors in the timing of their impact on progression. Subsequently, Haber et al. [30] examined both protective and risk factors on years to initiation of drinking, years from initiation to at-risk drinking, and years from at-risk drinking to AD in females. Risk factors included childhood psychiatric disorders (both externalizing and internalizing disorders), family risk history, and traumatic events; protective factors were dimensions of religion and spirituality. Results indicated that both risk and protective factors influenced initiation of alcohol use but that they were differentiated in their influence on the latter two developmental stages with protective factors slowing transition to at risk drinking and largely risk factors accelerating transition to AD. Unfortunately, few association studies have included later stages of remission and relapse in midlife in the course of alcohol use disorders. The current study addressed this need by examining onset of, remission from, and relapse to alcohol problem use through the 40’s and 50’s.

Specifically, the current study examined predictors associated with the duration of different stages in the progression of alcohol use in a sample of male veteran twins with AD history. After examining risk factors for age of first drink, the three successive transitions of onset, remission, and relapse were examined for both heavy drinking and AD. Thus, seven survival analysis models examined significant predictors of the time function, which permitted identification of risk and protective factors that accelerated or delayed the rates of transition through each distinct developmental period. The current study examined psychiatric variables, family history of AD, personality traits, alcohol expectancies, and drinking motives as possible predictors of transitions. Prediction from heavy drinking to remission was of particular interest for clinical treatment, as physicians and counselors could benefit from knowing which factors are most influential in supporting treatment success, especially if such predictors can be modified via therapy. We had the following general hypotheses, which are exploratory in nature but are based on patterns seen in past research on predictors of different aspects of alcohol use:

1. The same risk factors associated with initiation of drinking will also be associated with progression to heavy drinking and progression to AD (the three “onset” transitions).
2. Factors associated with remission from heavy drinking and remission from AD will be different from those predicting initiation and progression, with psychosocial factors being prominent influences.
3. Factors associated with relapse will be similar to those associated with onset and progression of heavy drinking and AD.

## Methods

### Participants

The participants in this study were twin veterans who were members of the Vietnam Era Twin (VET) Registry, although the twins were treated as individuals in the study. The VET Registry has been described in detail elsewhere [31–34], so only salient aspects will be described here. This national military twin registry comprises male twin pairs born between 1939 and 1957 in which both members served in the United States military between May 1965 and August 1975. In 1987, registry members completed a mailed questionnaire about their military experience, general health, marital and family history, etc. [31, 33] In 1992, a psychiatric interview was administered by telephone to twins who completed the questionnaire, assessing a range of psychiatric disorders, including alcohol and drug use disorders [34]. Information from the 1987 and 1992 assessments was used to design two offspring-of-twins studies that provided the current sample: one that began in 2000 focused on alcohol dependence history (Children of Alcoholics Study, COA [35]) and another that began in 2003 focused on drug dependence history (Twins as Parents Study, TAP [36, 37]). Equivalent procedures and identical assessments were used in both studies that permitted the planned combining of data across the two samples. In both the COA and TAP studies, eligible twin pairs were drawn from the VET Registry using an algorithm based on the following criteria: (a) both twins completed the 1987 and 1992 surveys; (b) at least one twin reported having children; and (c) at least one twin met criteria for a lifetime diagnosis of alcohol dependence (for COA Study) or drug dependence (for TAP Study), based on the Diagnostic and Statistical Manual, Third Edition, Revised (DSM-III-R [38]). This high-risk sampling plan identified 1062 VET Registry pairs, where cases with comorbid drug and alcohol histories represented the higher end of the substance dependence severity continuum. Both samples also included non-dependent cases with and without substance abuse histories to represent the lower end of the severity continuum. Although selection was based on pairs, each twin was individually contacted for participation, and twins were not excluded if a co-twin declined to participate. Concerted attempts were made to locate and enroll every individual selected for the studies. In the COA, 83% (n = 1295) of the individual twins were interviewed; while the comparable rate for TAP was 81% (n = 725). This resulted in 1774 individual veteran twin cases and 1769 cases met minimum data requirements for the current study. Information provided from previous interviews with the VET Registry twins was also combined with the COA/TAP collected data and used in data analyses. Thus, the predictor variables used in the analysis were collected at various timepoints and did not necessarily occur prior to the age of event; although we refer to predictors for increased or decreased time-to-event, it is important to note that the risk and protective factors are associated with, rather than causally related to, the speed of transition.

### Assessments

Procedures for obtaining verbal informed consent were approved by institutional review boards. Data collection was conducted by the Institute for Survey Research at Temple University. Interviewers, who were blind to the twin’s substance use history, used a computer-assisted telephone interview system with standardized interview questions and probes. After personal identifiers were removed by the VET Registry, data were released to the investigators. Interviews for the twin fathers in COA/TAP included a lifetime drinking history (LDH) assessment [39, 40] that was modified to include DSM, Fourth Edition (DSM-IV [41]) alcohol use disorder symptomatology. This assessment yields retrospective information about different phases of drinking across the life course, defined by the interviewee as a time when drinking frequency or quantity changed. To get information about their drinking life, the participants report starting and ending ages for each time period of different drinking pattern as well as other information about drinking during each phase including quantity/frequency of drinking and alcohol abuse and dependence symptomatology. These data provided the information we needed for the transitions of interest: age of onset for alcohol use and dependence diagnosis and ages for the changes in the patterns of drinking that indicate remission and relapse. Age of first AD was defined as the age at which DSM-IV criteria for AD were first met (i.e., yes to at least three of the DSM-IV criteria occurring within the same 12 months). The remission ages were derived from the age of the start of the first phase a given individual no longer met the AD criteria, and age of relapse was the age at the start of the first phase after remission where criteria were again met. The same rules were followed for heavy drinking age patterns, where heavy drinking was defined as a quantity-frequency index (QFI) score greater than or equal to 60 (quantity of drinks per occasion times the number of times drinking per month), which for men would be 14 drinks per week which is the cut-off for NIAAA’s [42] definition of “at risk/heavy” drinking. In survival analyses, individuals who do not meet criteria for a particular event (e.g., never reporting a change in drinking from AD diagnosis to remission) would be included as censored cases (indicating the event has not happened up to the individual’s current age). Studies investigating the reliability and validity of the LDH have supported its use to measure drinking patterns over time [39, 43, 44]. Family alcohol history was assessed by asking the men about their mother’s, father’s, and co-twin’s “excessive drinking” with a yes or no response. Sociodemographic covariates were collected in 2000 and included the veteran’s current age, highest educational attainment (in years), and income (in 19 categories).

Each veteran’s other lifetime psychiatric diagnostic information was obtained from their 1992 interview using DSM-III-R criteria. Nicotine dependence was used as a risk factor. Additionally, both externalizing and internalizing factors, which are dimensional measures used as alternatives to categorial diagnoses [45, 46] were created to index overall aspects of psychiatric risk. An externalizing risk variable, to index outward conflict, was created by the average of the z-scores for the symptom counts for antisocial personality disorder (ASPD) and other drug dependence (DD). An internalizing risk variable, to index internal distress, was created by the average of the z-scores for the symptoms counts for panic disorder, generalized anxiety disorder (GAD), posttraumatic stress disorder (PTSD), and major depression disorder (MDD). Although other mood and anxiety disorders also fall under the internalizing spectrum [45], other diagnoses were either not assessed or were uncommon (e.g. dysthymia).

Individual variable data, collected via a self-report mailed questionnaire in 2000, included the big five personality traits (extraversion, agreeableness, conscientiousness, openness, and neuroticism) assessed with the revised NEO Personality Inventory [47, 48], seven alcohol expectancies (risk and aggression, tension reduction, sociability, sexuality, liquid courage, cognitive and behavioral impairment, and self-perception) assessed with the Comprehensive Effects of Alcohol scale [49], and four drinking motives (social, coping, conformity, and enhancement) with the Drinking Motives Questionnaire [50]. Sample sizes for these individual variables are smaller than for the psychiatric variables as the mailed questionnaire was only returned by 77% (n = 1001 of 1295) of the COA sample and was not completed by the TAP sample. Missing data for various items and scales also led to smaller sample sizes for some variables.

### Data analysis

Survival analyses were completed in SAS [51] using Proc PHREG. The dependent variable in each of the seven stages was the number of years between successive events. The first transition was the number of years to the first full drink. Then onset, remission, and relapse transitions were examined for heavy drinking. Thus, the second transition was the period in years from the first drink to the start of the first heavy drinking period, the third transition was the number of years of heavy drinking to starting remission, and the fourth transition was the years in remission until relapse to heavy drinking levels. Then, we repeated examination of the same onset, remission, and relapse periods but with respect to AD diagnosis; that is, fifth was the number of years from the first drink to the start of the first phase the veteran was given an AD diagnosis; sixth, years from AD to the start of remission; and seventh, years in remission until relapse to next phase AD diagnosis was met. Only individuals who had experienced the prior event were included in the analysis for the next transition, where they either experienced the subsequent transition after a certain period of time or were a censored case (e.g., only if an individual had an age of first AD diagnosis were they included in the analysis for remission from AD).

The predictor variables included a range of demographic, psychiatric, and individual variables derived from various VET Registry assessments. The basic demographic variables were entered first in each model (age of interview, age of event, highest education level, and income). The demographic variables that were significant predictors of the time lapse variable were then also included in each subsequent survival analysis as covariates. Otherwise, first five different analyses for a transition stage were completed, each including one set of predictors: (a) the three psychiatric risk variables, (b) the three family history risk variables, (c) the five personality traits, (d) the four drinking motives, or (e) the seven alcohol expectancies. Once predictors were identified within each of the five domains, two across-domain survival analyses were completed for each transition stage to see which predictors remained significant across domains: one model included the significant psychiatric and family risk factors together (domains a and b; labeled “Clinical Risk”) and one included the significant individual predictor variables together (domains c, d, and e; labeled “Individual Predictors”). It is these across-domain model results that are presented here; the results from the within-domain models can be found in the Additional file 1: Table S1. Models were analyzed as described above to accommodate dataset limitations and to maximize power in sets of analyses. Specifically, because a subset of the sample completed the mailed survey that provided the data for the individual personality predictors (domains c, d, and e), these domains were run separately so sample size for the psychiatric predictors was not reduced. The hazard ratios presented in the tables for dichotomous variables indicate the risk of transition for those with the variable (e.g., a 1) as being x% of the risk for those without the variable, after controlling for the other variables in the model. For continuous variables, the interpretation changes, such that when subtracted from 1 and multiplied by 100, it gives the percent of change in risk for each one unit increase in the variable score [52, 53]. Thus, for onset and relapse transitions (e.g., the time from first drink to first AD), hazard ratios over 1 indicate a shorter time of transition (increased risk), while hazard ratios under 1 indicate a longer time of transition (protective delay). In contrast, for remission (e.g., time from AD to remission, where shorter times are better outcomes), hazard ratios under 1 indicate a risk factor and hazard ratios over 1 indicate a protective factor. To correct for the many comparisons made in the models, we present all findings significant at *p* < .05 in Table 2 but focus our discussion and explanation on the predictors significant with a stricter *p* < .01 level so as to not over-interpret effects.

## Results

### Sample characteristics

This sample of 1769 COA/TAP male-male twin veterans had a mean age of 51.8 years (*SD* = 2.9, range 43–63), had 13.7 (*SD* = 1.8) years of education on average, and had a mean household income of about $54,000 per year. This high-risk sample had a substantial family history of heavy alcohol use in that 49% reported that their father, 11% that their mother, and 48% that their twin brother had a history of excessive drinking. In terms of lifetime psychiatric history, 12% met criteria for PTSD, 11% met criteria for MDD, 5% met criteria for ASPD, 3% met criteria for GAD, and 2% met criteria for Panic Disorder. Concerning lifetime substance history, 56% met criteria for nicotine dependence, 47% for AD, and 22% for DD.

Table 1 reports the descriptive statistics for onset, remission, and relapse variables. Concerning the progression of alcohol use, 98.2% of the entire sample (1738 of 1769 cases) endorsed having a first full drink. Of those who initiated drinking, 74.5% (1295) had a period of heavy drinking, 72.0% (932) of heavy drinkers remitted, and 16.8% (157) of remitters relapsed back into heavy drinking. Of those who initiated drinking, 36.9% (641) became alcohol dependent, 81.7% (524) of those with AD remitted, and 13.0% (68) of remitters relapsed back into AD. Individuals who had not yet experienced an event (for example, the 31 men who had not yet had a full drink) are censored in survival analysis, with the logic that it is possible the individual will in the future experience the event but at his current age had not yet done so. The average number of years between the transitions ranged between 5.3 years (for first drink to first heavy drinking) and 11.5 years (for first heavy drinking to remission).

**Table 1:** Descriptive statistics (sample size, mean, standard deviation, minimum, and maximum) for age-at-event and years-between-event variables

Variable	N	Mean	SD	Min	Max
Age first drink	1738	16.3	2.9	1	42
Age of first HD	1295	21.1	5.8	12	55
Age of first remission from HD	932	32.0	8.9	17	56
Age of first relapse back to HD	157	37.5	8.2	20	54
Years between first drinking and HD	1293	5.3	5.6	0	38
Years between HD and remission	932	11.5	8.5	0	37
Years between remission and relapse back to HD	157	9.3	6.5	0	31
Age of first AD	641	24.6	7.9	12	54
Age of first remission	524	34.9	9.0	15	56
Age of first relapse	68	37.1	8.5	17	52
Years between first drinking and first AD	640	9.1	7.7	0	46
Years between first AD and first remission	524	11.2	8.1	0	36
Years between first remission and relapse	68	9.1	6.4	0	27

*Min* minimum, *Max* maximum; together these two values create the range. *HD* heavy drinking, quantified as QFI (quantity times frequency of drinking) greater than or equal to 60 drinks a month. *AD* alcohol dependence. One individual reported that the age of their first drink was at 1-year old, with others reporting other very young ages; missing data on other variables removed most of these participants from subsequent analyses, and age of first drink is not relevant for later transitions for those who do have later HD or AD data

### Hypothesis testing

Table 2 gives the hazard ratios for the risk and protective variables across all drinking transitions. Of note, the size of the reported effects, although significant, were generally small. As reminders, the table indicates level of significance with superscripts but only variables significant at the *p* < .01 level will be interpreted and results controlled for significant covariates as well as the age of the previous stage. The “Clinical Risk” model included all psychiatric and family history risk variables in one analysis (for each transition) and the “Individual Predictors” model included all personality, drinking motives, and expectancy variables. As stated above, hazard ratios greater than one reflect “risk” for onset and relapse outcomes but “protection” for remission outcomes.

**Table 2:** Hazard ratios from survival analyses predicting rate of progression to age of first drink, first heavy drinking, remission, and relapse, and age of first alcohol dependence, remission, and relapse

Model/variables	To first drink	Heavy drinking			Alcohol dependence		
		To first HD	To remission	To relapse	To first AD	To remission	To relapse
	1738 event, 31 censored	1293 event, 442 censored	932 event, 363 censored	157 event, 775 censored	640 event, 1904 censored	524 event, 117 censored	68 event, 456 censored
Model 1: Clinical risk	N = 1158	N = 1141	N = 1288	N = 675	N = 1113	N = 506	N = n/a
Psychiatric risk							
Nicotine dep	1.19**	1.30***	–	–	1.40**	–	–
Externalizing	1.41***	1.14***	0.89**	1.28^†^	1.31***	0.89^†^	–
Internalizing	–	1.06	1.16**	–	1.38***	–	–
Family history							
Father	1.13^†^	1.15^†^	–	–	1.30**	–	–
Mother	–	–	–	–	–	–	–
Twin	1.28***	1.39***	–	1.54^†^	2.22***	0.79^†^	–
Model 2: individual predictors	N = 925	N = 931	N = 715	N = 517	N = 900	N = 334	N = 279
Personality traits							
Extraversion	–	–	0.97***	–	–	–	0.95
Openness	1.01	–	–	1.05^†^	–	–	–
Neuroticism	0.98**	–	–	–	1.02^†^	–	–
Agreeableness	0.97**	1.00	1.03**	0.95^†^	0.99	1.03**	–
Conscientiousness	–	–	1.02**	–	–	–	–
Drinking motives							
Enhancement	1.02^†^	1.05***	0.97**	–	1.04**	–	–
Social	1.02	–	0.97**	1.10***	–	–	–
Coping	–	–	0.97**	–	1.05***	0.95***	1.15***
Conformity	–	–	1.05**	–	–	–	0.86**
Alcohol expectancies							
Risk and aggression	1.08***	1.09***	–	1.12	1.14***	–	–
Tension reduction	–	1.10**	0.99	–	–	–	–
Sociability	1.05	–	–	–	–	–	–
Sexuality	–	–	–	0.78^†^	–	–	–
Liquid courage	–	–	–	0.74**	–	–	–
Cog and Beh impair	0.96	0.96	1.14***	–	–	1.00	–
Self-perception	–	–	–	–	1.07	–	–

^†^*p* < .05; **p ≤ .01; ***p ≤ .001

*ns* not significant. Variable was included in the model as it was a significant predictor in earlier models, but was not significant in the final model

– Not included in the model as the variable was not a significant predictor of the outcome in a previous model where only the variables within the specific subdomain were included at the same time

*HD* heavy drinking, *AD* alcohol dependence. *Externalizing* antisocial personality disorder and drug dependence. *Internalizing* Major depression, panic, post-traumatic stress, and generalized anxiety disorders. *Cog and Beh Impair* cognitive and behavioral impairment

All models also included a control for the previous transition and demographic variables (age, income, and education level). Missing data for ages or other variables creates slight differences in numbers of cases counted towards events across models

Hypothesis 1 examined the consistency of factors predicting the three transitions of onset of alcohol use problems: years to first drink and subsequent years to either heavy drinking or AD. Four risk factors were identified as being associated with accelerating all three onset variables (hazard ratios greater than 1.0): nicotine dependence (ORs = 1.19–1.40), externalizing disorders (ORs = 1.14–1.41), cotwin drinking history (ORs = 1.28–2.22), and the alcohol expectancy of risk and aggression (ORs = 1.08–1.14). Another risk factor, the drinking motive of enhancement, predicted both earlier onset of heavy drinking and of alcohol dependence (ORs = 1.05, 1.04). Significant consistency was evident across these three transitions in risk, but four single-stage specific risk and protective factors were also identified. For drinking onset, both agreeableness (OR = 0.97) and neuroticism (OR = 0.98) were protective factors that delayed years to first drink. The unique association for years from first drink to onset of heavy drinking was the risk factor of a tension reduction expectancy (OR = 1.10). For years from first drink to first AD, the specific risks of internalizing disorders (OR = 1.38), father drinking history (OR = 1.30), and coping drinking motives (OR = 1.05) were related to quicker transition.

Hypothesis 2 evaluated factors associated with remission in contrast to the various transitions of progression towards higher levels of use. Those factors that were related to accelerated remission were considered protective factors; those connected to delaying remission were risk factors. For years to remission of heavy drinking, significant risk for delayed remission came from externalizing disorders (OR = 0.89), the personality trait of extraversion (OR = 0.97), and the three drinking motives of enhancement (OR = 0.97), social (OR = 0.97), and coping (OR = 0.97) while significant protective factors accelerating remission were internalizing disorders (OR = 1.16), the traits of agreeableness (OR = 1.03) and conscientiousness (OR = 1.02), the drinking motive of conformity (OR = 1.05), and the expectancy of cognitive and behavioral impairment (OR = 1.14). Many fewer significant predictors of years to remission of AD were found but they were the same as the predictors of remission from heavy drinking: coping motives (OR = 0.95) again delayed remission and agreeableness again accelerated remission (OR = 1.03); externalizing was a risk factor only at the *p* < 0.05 level, although with the same effect size as for heavy drinking. In comparing predictors across remission and onset transitions, there were seven significant predictors of either remission variable that were unique and not shared with the onset risk and protective factors, while three factors were consistent across the two remission transitions and also significant for at least one onset transition (the risk factor of externalizing, the risk factor of coping motives, and the protective factor of agreeableness).

Hypothesis 3 compared the predictors of onset transitions (first drink, heavy drinking, and AD) with the factors associated with relapse after remission. Few factors predicted remission with a significant effect: only the risk factor of social drinking motives (OR = 1.10) and the protective factor of expectancy of liquid courage (OR = 0.74) for heaving drinking and the risk factor of coping motive (OR = 1.15) and protective factor of conformity motives (OR = 0.86) for AD. Of these, only two worked in similar ways during other transitions: social motives had also delayed remission of heavy drinking and coping motives had also accelerated initiation and slowed remission of AD. Surprisingly, the major onset risk factors of externalizing and cotwin drinking history predicted relapse of AD and predicted relapse of heaving drinking only at a *p* < .05 level.

## Discussion

Using conditional survival analysis, the current study examined the amount of time between transitions in the progression to initiation of drinking, heavy drinking, and diagnosis of alcohol dependence, and then further examined two later transitions, remission and relapse, thus characterizing the natural course of an alcohol use trajectory. This unique, time-based research method examined the predictors identified in current alcohol use disorder literature and their consistency in predicting progression from one transition to the next. Unlike other event-based research methods, this method examined each transition independent of earlier effects, thus providing a more precise characterization of factors relevant to that timeframe alone. Results will be discussed in terms of the hypotheses.

Hypothesis 1 was supported in that there was consistency across predictors of the three onset transitions (years to first drink, years from initiation to first heavy drinking, and years from initiation to first AD) with four risk factors associated with acceleration to all three onset transitions and one consistent across the two more problematic drinking transitions. Of note, however, is that some overlap in significant predictors may not be surprising because some of the same cases would be included, albeit with potentially different years-to-event, in the heavy drinking and AD models (for the 1295 heavy drinking cases, 620 of them were also in the 641 heavy drinking cases, with a correlation between age of onset for heavy drinking and age of onset for AD of 0.51). The first two predictors were psychiatric variables that are comorbid with alcohol use: nicotine dependence and externalizing disorders. Research supports the idea that not only is there overlap between the occurrence of problematic use of nicotine and alcohol, but that common genetic underpinnings underlie risk for nicotine, alcohol, and other drug use [54, 55]. In fact, the entire spectrum of externalizing disorders, including alcohol and drug use but also other aggressive and disinhibitory disorders and personality traits, has been shown to share genetic and biological vulnerability [56, 57]. Past research has also found that externalizing problems predicted subsequent alcohol use in adolescence and early adulthood [58, 59].

The third predictor was a family history variable: co-twin’s history of drinking. A substantial literature documents the role of family history in alcohol use [1, 5]. These outcome patterns likely arise from common variance shared by alcohol dependence and concomitant disorders that is substantially genetic in nature, and therefore is shared by twins, is stable across time, and is transmitted to offspring as familial resemblance [19, 60, 61].

Fourth, and turning to psychosocial influences, current findings implicated the alcohol expectancy of risk and aggression as a factor accelerating onset progression. In a sample of men, Patrick and Schulenburg [62] found that individuals who expect that alcohol use will increase their dominance and toughness were likely to start using or increase their use more quickly than men without this expectation. Risky behavior is not always viewed as a negative side effect of drinking, and it appears that those who favor risk and aggression will more quickly progress to alcohol use onset.

Fifth, current findings implicated the drinking motive of enhancement as associated with accelerated progression toward heavy drinking and AD onset transitions. Enhancement motives have been shown to predict increased drinking quantity and drinking misuse from adolescence to middle-adulthood [63]. Given that the average age of first transitions to heavy drinking and AD is in adolescence and early adulthood, it is not surprising that positive feelings of enhancement and enjoyment predict accelerated transition to greater alcohol use.

Taken together, these findings demonstrate the consistency of five well-recognized onset predictors of accelerated progression toward three key onset transitions: years to first drink, years from initiation to heavy drinking, and years from initiation to first AD. These results confirm and clarify current models of problem alcohol use etiology using a more precise methodology, and results at least partially confirm hypothesis 1 that onset risk factors are similar across the three onset variables. However, there were some stage-specific risk and protective factors as well. For example, the alcohol expectancy of tension reduction was related to accelerated progression to heavy drinking but not initiation or AD. Patrick and Schulenburg [62] used growth models to show that adolescent binge drinking was related to avoiding boredom and that early adult binge drinking was related to getting away from one’s problems, findings that suggest a tension reduction expectation specific to heavy drinking. Other transition-specific factors were the protective personality factors of agreeableness and neuroticism for delayed first drink; although research suggests [1, 21, 64], as discussed further below, that agreeableness should be protective against alcohol use, neuroticism is generally found to be a risk factor for drinking and not a protective factor. Further research could investigate the effect of personality on various aspects of drinking onset to examine potential differential prediction.

Hypothesis 2, that the predictors of delayed remission would be different than the predictors of accelerated onset and would likely be more psychosocial in nature, was supported. Of the five onset risk factors identified above, only externalizing disorders and the drinking motive of enhancement affected remission from heavy drinking while none affected remission from AD. There were a few other risk factors that delayed remission that were not consistent onset risk factors: namely the personality trait of extraversion and the social and coping drinking motives.

Additionally, five other variables were identified as protective factors that were not significantly related to onset transitions. Thus, it was other aspects of our measured domains that had important connections to a quicker decrease in alcohol use. First, in terms of clinical variables, internalizing disorders speeded progress toward remission of heavy drinking. Internalizing disorders are well-substantiated risk factors that are central to the internalizing, negative affect pathway to alcohol use [4, 65]. Thus, it was surprising to find internalizing disorders associated with shortened remission. The currently observed effect may be masked in standard association studies because later milestones necessarily include influences on earlier milestones which can swamp proximal effects. Given the precision of the current model, it appears that after one achieves a heavy drinking pattern, the sedating effect of alcohol may dissipate while leaving an increasing depressive effect that in turn prompts a desire for remission. Although reasonable, this is admittedly an unusual finding. Some researchers have found links between depressive disorder and more excessive drinking trajectories as opposed to recovering drinkers [3], while others have found no systematic link between episodes of emotional dysregulation and episodes of alcohol problem use, as to whether the emotional regulation issues preceded, co-occurred, or followed the alcohol use [66] and no link between having (or not having) depression and anxiety disorders and remission from alcohol dependence [15]. The inconsistencies here deserve further study and investigation with respect to potential moderators or interaction effects in the internalizing—alcohol use relationship.

The other four factors related to accelerated remission were psychosocial in nature. Beginning with personality, agreeableness was found to accelerate remission from heavy drinking and AD and conscientiousness was found to accelerate remission from heavy drinking. The general personality profile associated with alcohol dependence is low agreeableness, low conscientiousness (related to impulsivity and disinhibition), and high neuroticism [1, 21, 64], a pattern otherwise known as (reversed) stability [67]. The increased maturity seen in personality, based on these traits, seen in early adulthood corresponds to a decrease in general prevalence of alcohol problem use and it is suggested this maturation continues through later life [68]. Current findings for remission are consistent with this profile.

Remission from heavy drinking was also accelerated by the drinking motive of conformity. Remission generally occurs later in one’s drinking career, whereas enhancement (enjoyment) motives may be most apparent at earlier ages and stages (as it was with onset of heavy drinking and AD in our sample), drinking motives change over time [50]. In the current sample, relapse occurred, on average, at age 37. It may be that, in midlife, greater maturity is associated with greater interpersonal responsiveness (“conformity”) that may, in turn, encourage remission (as seen here). Similarly, concerning alcohol expectancies, the anticipation of cognitive and behavioral impairment was also found to be related to accelerated remission from heavy drinking. It may be that a good dose of “reality” is important for change, and awareness of the negative impact of excessive alcohol use on one’s mental functioning and behavior may, in turn, accelerate remission [69]. The impact of these personality, motive, and expectancy variables on remission is noteworthy because psychosocial factors are much more amenable to treatment compared to clinical disorders. The current findings suggest that treatment may be enhanced by interventions that encourage development of prosocial behaviors including agreeableness, conscientious behavior, social support, and awareness of risks of alcohol impairment.

Hypothesis 3 turned to the predictors of relapse, with the hypothesis that the factors associated with relapse would be similar to those associated with onset and progression of heavy drinking and AD finding little support. In the current data, after remission had been achieved, findings implicated that few, if any, of the consistent risk factors for onset were also risks for the relapse transitions. Externalizing disorders and co-twin history were surprisingly not strongly predictive, being significant only at the *p* < .05 level, although sample size (as discussed below) was problematic for relapse transitions and could have affected significance levels. The other consistent onset risk factors of cotwin nicotine dependence, risk and aggression expectancies, and enhancement motives were also not significant for relapse. Instead, there were four predictors for relapse transitions that were not consistent onset predictors.

One of these was the risk factor of social drinking movies for accelerated relapse of heavy drinking. It appears that social drinking motives are not what is needed when one is in remission from an alcohol use disorder. The social aspect of drinking is potentially central to the ongoing debate between those who argue that moderation of drinking is the best path to recovery and Alcoholics Anonymous (AA) that insists on complete abstention from alcohol use [70]. Social needs are a part of the human experience, but when a person’s social circle uses drinking as a central activity, it can be difficult to remain abstinent. An essential part of the AA program is the required attendance at meetings with fellow sober and clean AA members who are in recovery [71]. Given that research has found involvement in AA and 12-step programs is a positive predictor of recovery trajectories [2, 72], the role of social structure and support in sustaining recovery is evident. Thus, the tendency toward social drinking may be problematic in this situation, and this effect is confirmed in these data.

The factor that was found to be significantly related to accelerated relapse to AD was the drinking motive of coping. The results related to the risk factor of coping were interesting, in that coping was a consistent risk factor for all AD transitions (a trigger for onset, a delay in remission, and a speeded relapse) though not a significant predictor for most heaving drinking transitions (except remission). This finding underscores the greater risk of “coping by drinking” for those who have met criteria for AD and who are probably later in their alcohol use career. Other research has also found that drinking to avoid problems was related to heavier drinking in later early adulthood (age 22–30) [62]. The current data suggests a close relationship between alcohol dependence and alcohol coping.

For protective factors against relapse, the expectancy of liquid courage for heavy drinking and the motive of conformity for AD were fairly unique to these transitions. Of these, recall that a conformity motive was related to accelerated remission from heavy drinking. It may be that greater age and maturity increases interpersonal responsiveness (“conformity”) that, in turn, encourages both remission (from heavy drinking) and the delay of relapse (back to AD).

In addition to the support found for hypotheses 1 and 2 and lack of support for hypothesis 3, another comparison in the current data could be interesting. Although we thought that more psychosocial variables would affect remission more than onset and relapse (part of hypothesis 2), which has some support, the data also suggested that there were more individual psychosocial predictors for first drink and heavy drinking transitions than for AD transitions. It is possible that more individual or cultural motives and expectancies could drive drinking frequency and quantity while more physiological components of addiction (e.g., tolerance, withdrawal, craving) may be predictive for diagnosable drinking issues. The conclusion here is tentative, given the smaller sample size for the AD transitions, but may be of potential interest for further investigation.

This study was not without its limitations. The current sample included only men so it is unknown if the same variables would be associated with transitions in women: Research has suggested that late-life transitions of alcohol use may differ in timing for women compared to men [73]. The sample was also a veteran sample, thus also limiting generalizability to the extent that veterans differ from the general population (e.g., higher rates of PTSD or other mental health issues) [74, 75]. The retrospective nature of the drinking assessment is limited by memory issues of past drinking patterns and behaviors. However, as cited above, research studies have shown the LDH to be a reliable and valid way to derive drinking history. The retrospective design and VET Registry assessment timeline provided a lot of information across many variables of interest. A limitation of this assessment protocol, however, was that the predictor variables were not always assessed prior to the age of initiation, remission, or relapse. A time-varying approach to the predictors would be useful for future research, where the exact timing of events could be delineated for each individual to more clearly indicate prediction of a transition. Sample size was also an issue for the relapse variables, where many men had not (yet) experienced the transition. The number of men who had experienced relapse of AD was especially small (N = 68), and this could be one reason we find so few significant risk and protective factors for relapse of AD as compared to relapse of heavy drinking. Additionally, the limited data for the individual predictors from the self-report mailed questionnaire made comparisons across domains problematic. The use of a large number of predictor variables could also be an issue. Although the point of the analyses was to examine many potential risk and protective factors, it is possible that Type I errors occurred amongst the many significance values. Effect sizes were small, as noted previously, although effects for clinical risk variables were generally stronger than psychosocial variables. We focused on results that were consistent across transitions (when possible) and on effects significant at a more stringent probability level. Additionally, there are other potential risk and protective factors that were not examined in the current analyses, including marital stability and religious variables, but it was necessary to limit our range of inquiry.

Using time-based survival analyses to identify faster or slower progression to key developmental transitions in alcohol use, the current study examined domains of risk identified in current alcohol problem use etiology literature. Results confirmed that known risk factors were associated with faster progression toward three key onset transitions and then extended examination to subsequent remission and then later relapse. Further research should continue the effort to identify the predictors that hasten or slow the progression of drinking throughout an alcohol use career. The study of remission and relapse may be particularly informative to clinicians and others involved in the treatment of substance disorders. Importantly, the role of psychosocial variables as predictors of remission and the delay of relapse may provide empirically-identified avenues of treatment intervention that may inform developing treatment programs.

## Supplementary information

**Additional file 1: Table S1**. Hazard ratios from survival analyses predicting rate of progression through stages of alcohol use, where predictors were entered in domain groups (e.g., the three psychiatric risk factors together in one model; the five personality traits together in one model). Variables that were significant in these models were included in the models discussed in the manuscript and shown in Table 2 in the paper.

## Data Availability

The datasets generated and/or analyzed during the current study are not publicly available because the data are part of a larger registry and projects for which data analyses are still ongoing and do not solely belong to the authors of this paper.

## Abbreviations

AA: Alcoholics Anonymous
AD: Alcohol dependence
AUD: Alcohol use disorders
ASPD: Antisocial personality disorder
COA: Children of Alcoholics study
DD: Drug dependence
DSM: Diagnostic and Statistical Manual
GAD: Generalized anxiety disorder
LDH: Lifetime Drinking History
MDD: Major depression disorder
N: Sample size
NIAAA: National Institute for Alcohol Abuse and Alcoholism
OR: Odds ratio/hazard ratio
PTSD: Posttraumatic stress disorder
QFI: Quantity-frequency index
SES: Socioeconomic status
TAP: Twins as Parents study
VET Registry: Vietnam Era Twin Registry

## References

[1] Chassin L, Flora DB, King KM (2004). Trajectories of alcohol and drug use and dependence from adolescence to adulthood: the effects of familial alcoholism and personality. J Abnorm Psychol.

[2] Cranford JA, Krentzman AR, Mowbray O, Robinson EAR (2014). Trajectories of alcohol use over time among adults with alcohol dependence. Addict Behav.

[3] Fuehrlein BS, Kachadourian LK, DeVylder EK, Trevisan LA, Potenza MN, Krystal JH (2018). Trajectories of alcohol consumption in US military veterans: results from the national health and resilience in veterans study. Am J Addict.

[4] Sher KJ, Grekin ER, Williams NA (2005). The development of alcohol use disorders. Annu Rev Clin Psycho.

[5] Wichers M, Gillespie NA, Kendler KS (2013). Genetic and environmental predictors of latent trajectories of alcohol use from adolescence to adulthood: a male twin study. Alcohol Clin Exp Res.

[6] Zucker RA, Cicchetti D, Cohen DJ (2006). Alcohol use and the alcohol use disorders: a developmental-biopsychosocial systems formulation covering the life course. Developmental psychopathology, vol 3: risk, disorder, and adaptation.

[7] Jacob T, Koenig LB, Howell DN, Wood PK, Haber JR (2009). Drinking trajectories from adolescence to the fifties among alcohol-dependent men. J Stud Alcohol Drugs.

[8] Jacob T, Blonigen DM, Hubel K, Wood PK, Haber JR (2012). Drinking course through midlife based on diagnostic versus quantity-frequency indices. Alcohol Clin Exp Res.

[9] Fitzgerald HE, Puttler LI (2018). (2018) Alcohol use disorders: a developmental science approach to etiology.

[10] National Institute on Alcohol Abuse and Alcoholism. Five year strategic plan, 2007–2011: alcohol across the lifespan. 2006. https://pubs.niaaa.nih.gov/publications/StrategicPlan/NIAAASTRATEGICPLAN.htm.

[11] National Institute on Alcohol Abuse and Alcoholism Strategic Plan 2017–2021. https://www.niaaa.nih.gov/sites/default/files/StrategicPlan_NIAAA_optimized_2017-2020.pdf.

[12] McCutcheon VV, Grant JD, Heath AC, Bucholz KK, Sartor CE, Nelson EC (2012). Environmental influences predominate in remission from alcohol use disorder in young adult twins. Psychol Med.

[13] Krentzman AR, Cranford JA, Robinson EAR (2015). Long-term increases in purpose in life are associated with remission from alcohol dependence. Alcohol Treat Q.

[14] Moos RH, Brennan PL, Schutte KK, Moos BS (2010). Older adults’ health and late-life drinking patterns: a 20-year perspective. Aging Ment Health.

[15] Lopez-Quintero C, Hasin DS, Perez de los Cobos J, Pines A, Wang S, Grant BF, Blanco C (2010). Probability and predictors of remission from life-time nicotine, alcohol, cannabis or cocaine dependence: results from the National Epidemiological Survey on Alcohol and Related Conditions. Addition.

[16] Vaillant GE (2003). A 60-year follow-up of alcoholic men. Addiction.

[17] Tuithof M, ten Have M, van den Brink W, Vollebergh W, de Graaf R (2014). Alcohol consumption and symptoms as predictors for relapse of DSM-5 alcohol use disorder. Drug Alcohol Depen.

[18] Schutte K, Nichols K, Brennan P, Moos R (2003). A ten-year follow-up of older former problem drinkers: risk of relapse and implications of successfully sustained remission. J Stud Alcohol Drugs.

[19] McGue M, Iacono WG, Legrand LN, Elkins I (2001). Origins and consequences of age at first drink. II. Familial risk and heritability. Alcohol Clin Exp Res.

[20] Poulton R, Caspi A, Milne BJ, Thompson WM, Taylor A, Sears MR, Moffitt TEL (2002). Association between children’s experience of socioeconomic disadvantage and adult health: a life-course study. Lancet.

[21] Kotov R, Gamez W, Schmidt F, Watson D (2010). Linking “big” personality traits to anxiety, depressive, and substance use disorders: a meta-analysis. Psychol Bull.

[22] Willinger U, Lenzinger E, Hornik K, Fischer G, Schönbeck G, Aschauer HN, Meszaros K (2002). Anxiety as a predictor of relapse in detoxified alcohol-dependent patients. Alcohol Alcohol.

[23] Walter M, Gerhard U, Duersteler-MacFarland KM, Weihers HG, Boening J, Wiesbeck GA (2006). Social factors but not stress-coping styles predict relapse in detoxified alcoholics. Neuropschobiology.

[24] Vergés A, Jackson KM, Bucholz KK, Grant JD, Trull TJ, Wood PK, Sher KJ (2012). Deconstructing the age-prevalence curve of alcohol dependence: why “maturing out” is only a small piece of the puzzle. J Abnorm Psychol.

[25] Brennan PL, Nichols KA, Moos RH (2002). Long-term use of VA mental health services by older patients with substance use disorders. Psychiatr Serv.

[26] Suter M, Strik W, Moggi F (2011). Depressive symptoms as a predictor of alcohol relapse after residential treatment programs for alcohol use disorder. J Subst Abuse Treat.

[27] Haber JR, Grant JD, Jacob TJ, Koenig LB, Heath A (2012). Alcohol milestones, risk factors, and religion/spirituality in young adult women. J Stud Alcohol Drugs.

[28] Koenig HG, McCullough ME, Larson DB (2001). Handbook of religion and health.

[29] Sartor CE, Lynskey MT, Heath AC, Jacob T, True W (2007). The role of childhood risk factors in initiation of alcohol use and progression to alcohol dependence. Addiction.

[30] Haber JR, Grant JD, Sartor CE, Koenig LB, Heath A, Jacob T (2013). Religion/spirituality, risk, and the development of alcohol dependence in female twins. Psychol Addict Behav.

[31] Eisen S, True W, Goldberg J, Henderson W, Robinette C (1987). The Vietnam Era Twin (VET) registry: method of construction. Acta Genet Med Gemellol Twin Res.

[32] Goldberg J, True W, Eisen S, Henderson W, Robinette C (1987). The Vietnam era twin (VET) registry: ascertainment bias. Acta Genet Med Gemellol Twin Res.

[33] Henderson WG, Eisen S, Goldberg J, True WR, Barnes JE, Vitek ME (1990). The Vietnam Era Twin Registry: a resource for medical research. Public Health Rep.

[34] Tsuang MT, Lyons MJ, Eisen SA, Goldberg J, True W, Lin N, Meyer JM, Toomey R, Faraone SV, Eaves L (1996). Genetic influences on DSM-III-R drug abuse and dependence: a study of 3,372 twin pairs. Am J Med Genet.

[35] Jacob T, Waterman B, Heath A, True W, Bucholz KK, Haber R, Scherrer J, Fu Q (2003). Genetic and environmental effects on offspring alcoholism: new insights using an offspring twins design. Arch Gen Psychiatry.

[36] Duncan AE, Sartor CE, Scherrer JF, Grant JD, Heath AC, Nelson EC, Jacob T, Bucholz KK (2008). The association between cannabis abuse and dependence and childhood physical and sexual abuse: evidence from an offspring of twins design. Addiction.

[37] Scherrer JF, Grant JD, Duncan AE, Pan H, Waterman B, Jacob T, Haber JR, True WR, Heath AC, Bucholz KK (2008). Measured environmental contributions to cannabis abuse/dependence in an offspring of twins design. Addict Behav.

[38] American Psychiatric Association (1987). Diagnostic and statistical manual of mental disorders (DSM-III-R).

[39] Skinner HA, Sheu WJ (1982). Reliability of alcohol use indices: the lifetime drinking history and MAST. J Stud Alcohol.

[40] Jacob T (1998). Modified lifetime drinking history. Unpublished measure.

[41] American Psychiatric Association (1994). Diagnostic and statistical manual of mental disorders (DSM-IV).

[42] National Institute on Alcohol Abuse and Alcoholism. Rethinking drinking: Alcohol and your health. (nd) https://www.rethinkingdrinking.niaaa.nih.gov/How-much-is-too-much/Is-your-drinking-pattern-risky/Whats-At-Risk-Or-Heavy-Drinking.aspx.

[43] Jacob T, Seilhamer RA, Bargiel K, Howell DN (2006). Reliability of lifetime drinking history among alcohol dependent men. Psychol Addict Behav.

[44] Koenig LB, Jacob TJ, Haber JR (2009). Validity of the lifetime drinking history: a comparison of retrospective and prospective quantity-frequency measures. J Stud Alcohol Drugs.

[45] Krueger RF (1999). The structure of common mental disorders. Arch Gen Psychiatry.

[46] Krueger RF, Markon KE, Patrick CJ, Iacono WG (2005). Externalizing psychopathology in adulthood: a dimensional-spectrum conceptualization and its implications for DSM-V. J Abnorm Psychol.

[47] Costa PT, McCrae RR (1992). Revised NEO personality inventory (NEO-PI–R) and the NEO five-factor inventory (NEO-FFI) professional manual.

[48] McCrae RR, John OP (1992). An introduction to the five-factor model and its applications. J Personal.

[49] Fromme K, Stroot EA, Kaplan D (1993). Comprehensive effects of alcohol: development and psychometric assessment of a new expectancy questionnaire. Psychol Assess.

[50] Cooper ML (1994). Motivations for alcohol use among adolescents: development and validation of a four-factor model. Psychol Assess.

[51] SAS Institute Inc (2006). SAS Statistical Software. Version 9.

[52] Allison PD (1995). Survival analysis using SAS: a practical guide.

[53] Kleinbaum DG, Klein M (2005). Survival analysis: a self-learning text.

[54] Kendler KS, Myers J, Prescott CA (2007). Specificity of genetic and environmental risk factors for symptoms of cannabis, cocaine, alcohol, caffeine, and nicotine dependence. Arch Gen Psychiatry.

[55] Richmond-Rakerd LS, Slutske WS, Lynskey MT, Agrawal A, Madden PF, Bucholz KK, Heath AC, Statham DJ, Martin NG (2016). Age at first use and later substance use disorder: shared genetic and environmental pathways for nicotine, alcohol, and cannabis. J Abnorm Psychol.

[56] Iacono WG, Malone SM, McGue M (2008). Behavioral disinhibition and the development of early-onset addiction: common and specific influences. Ann Rev Clin Psychol.

[57] Sadeh N, Spielberg JM, Logue MW, Hayes JP, Wolf EJ, McGlinchey RE, Milberg WP, Schichman SA, Stone A, Miller MW (2018). Linking genes, circuits, and behavior: network connectivity as a novel endophenotype of externalizing. Psychol Med.

[58] Edwards AC, Gardner CO, Hickman M, Kendler KS (2006). A prospective longitudinal model predicting early adult alcohol problems: evidence for a robust externalizing pathway. Psychol Med.

[59] Farmer RF, Gau JM, Seeley JR, Kosty DB, Sher KJ, Lewinsohn PM (2016). Internalizing and externalizing disorders as predictors of alcohol use disorder onset during three developmental periods. Drug Alcohol Depend.

[60] Kendler KS, Prescott CA (2006). Genes, environment, and psychopathology: understanding the causes of psychiatric and substance use disorders.

[61] Verhulst B, Neale MC, Kendler KS (2015). The heritability of alcohol use disorders: a meta-analysis of twin and adoption studies. Psychol Med.

[62] Patrick ME, Schulenburg JE (2011). How trajectories of reasons for alcohol use related to trajectories of binge drinking: national panel data spanning late adolescence to early adulthood. Dev Psychol.

[63] Patrick ME, Wray-Lake L, Finlay AK, Maggs JL (2010). The long arm of expectancies: adolescent alcohol expectancies predict adult alcohol use. Alcohol Alcohol.

[64] Sleep CE, Hyatt CS, Lamkin J, Maples-Keller JL, Miller JD (2018). Examining the relations among the DSM–5 alternative model of personality, the five factor model, and externalizing and internalizing behavior. Personal Disord.

[65] Hussong AM, Jones DJ, Stein GL, Baucom DH, Boeding A (2011). An internalizing pathway to alcohol use and disorder. Psychol Addict Behav.

[66] Farmer RF, Seeley JR, Kosty DB, Gau JM, Duncan SC, Sher KJ, Lewinsohn PM (2017). No reliable evidence that emotional disorders are proximal antecedents, concomitants, or short-term consequences of first episode alcohol use disorders in a representative community sample. J Stud Alcohol Drugs.

[67] DeYoung CG, Peterson JB, Higgins DM (2002). Higher-order factors of the big five predict conformity: are there neuroses of health?. Personal Individ Differ.

[68] Sher KJ, Littlefield A, Lee M, Fitzgerald HE, Puttler LI (2018). Personality processes related to the development and resolution of alcohol use disorders. Alcohol use disorders: a developmental science approach to etiology.

[69] Goldman MS, Del Boca FK, Darkes J, Leonard KE, Blane HT (1999). Alcohol expectancy theory: the application of cognitive neuroscience. Psychological theories of drinking and alcoholism.

[70] Anonymous A (2001). Alcoholics Anonymous big book.

[71] Gossop M, Stewart D, Marsden J (2008). Attendance at Narcotics Anonymous and Alcoholics Anonymous meetings, frequency of attendance and substance use outcomes after residential treatment for drug dependence: a 5-year follow-up study. Addiction.

[72] Witbrodt J, Mertens J, Kaskutas LA, Bond J, Chi F, Weisner C (2012). Do 12-step meeting attendance trajectories over 9 years predict abstinence?. J Subst Abuse Treat.

[73] Brennan PL, Schutte KK, Moos BS, Moos RH (2011). Twenty-year alcohol-consumption and drinking-problem trajectories of older men and women. J Stud Alcohol Drugs.

[74] Dohrenwend BP, Turner JB, Turse NA, Adams BG, Koenen KC, Marshall R (2006). The psychological risks of Vietnam for U.S. veterans: a revisit with new data and methods. Science.

[75] Jordan BK, Schlenger WE, Hough R, Kulka RA, Weiss D (1991). Lifetime and current prevalence of specific psychiatric disorders among Vietnam veterans and controls. Arch Gen Psychiatry.

