# Supplementary material for: Transitions in alcohol use over time: a survival analysis
Source: BMC Psychol. 2020 Nov 3;8:115. doi: 10.1186/s40359-020-00479-1 (PMC7607670; doi:10.1186/s40359-020-00479-1)
Supplement: Supplementary file 1 — Additional file 1: Table S1. Hazard ratios from survival analyses predicting rate of progression through stages of alcohol use, where predictors were entered in domain groups (e.g., the three psychiatric risk factors together in one model; the five personality traits together in one model). Variables that were significant in these models were included in the models discussed in the manuscript and shown in Table 2 in the paper. [file 40359_2020_479_MOESM1_ESM.docx]

Additional file: Table S1

*Hazard ratios from survival analyses predicting rate of progression through stages of alcohol use, where predictors were entered in domain groups (e.g., the three psychiatric risk factors together in one model; the five personality traits together in one model). Variables that were significant in these models were included in the models discussed in the manuscript and shown in Table 2 in the paper.*

|  |  |  |  |  | | |  |  | | |
| --- | --- | --- | --- | --- | --- | --- | --- | --- | --- | --- |
|  | To First Drink |  |  | Heavy Drinking | | |  | Alcohol Dependence | | |
|  |  |  |  | To first HD | To remission | To relapse |  | To first AD | To remission | To relapse |
|  |  |  |  |  |  |  |  |  |  |  |
|  |  |  |  |  |  |  |  |  |  |  |
| Model/Variables | *1738 event, 31 censored* |  |  | *1293 event, 442 censored* | *932 event, 363 censored* | *157 event, 775 censored* |  | *640 event, 1904 censored* | *524 event, 117 censored* | *68*  *event, 456 censored* |
|  |  |  |  |  |  |  |  |  |  |  |
|  |  |  |  |  |  |  |  |  |  |  |
|  |  |  |  |  |  |  |  |  |  |  |
| *Psychiatric Risk* | *N=1758* |  |  | *N=1726* | *N=1288* | *N=928* |  | *N=1673* | *N=625* | *N=516* |
| Nicotine dep | 1.32** |  |  | 1.41** | 0.98 | 1.03 |  | 1.46** | 1.13 | 0.93 |
| Externalizing | 1.47** |  |  | 1.17** | 0.89** | 1.24* |  | 1.37** | 0.85** | 1.17 |
| Internalizing | 0.99 |  |  | 1.11** | 1.17** | 1.11 |  | 1.46** | 1.06 | 1.25 |
|  |  |  |  |  |  |  |  |  |  |  |
|  |  |  |  |  |  |  |  |  |  |  |
| *Family History* | *N=1153* |  |  | *N=1136* | *N=891* | *N=648* |  | *N=1108* | *N=482* | *N=398* |
| Father | 1.13* |  |  | 1.19* | 0.94 | 0.93 |  | 1.41** | 0.90 | 0.94 |
| Mother | 1.19 |  |  | 0.95 | 1.02 | 1.21 |  | 1.12 | 0.99 | 0.42 |
| Twin | 1.35** |  |  | 1.48** | 0.87 | 1.56* |  | 2.54** | 0.79* | 1.44 |
|  |  |  |  |  |  |  |  |  |  |  |
|  |  |  |  |  |  |  |  |  |  |  |
| *Personality traits* | *N=988* |  |  | *N=967* | *N=742* | *N=539* |  | *N=936* | *N=344* | *N=286* |
| Extraversion | 1.00 |  |  | 1.03 | 0.98** | 0.98 |  | 1.00 | 0.99 | 0.92* |
| Openness | 1.02* |  |  | 1.01 | 1.01 | 1.06* |  | 1.01 | 1.00 | 1.03 |
| Neuroticism | 0.99* |  |  | 0.99 | 1.01 | 0.99 |  | 1.04** | 0.99 | 1.00 |
| Agreeableness | 0.96** |  |  | 0.98* | 1.03** | 0.95* |  | 0.96** | 1.04** | 1.02 |
| Conscientiousness | 0.99 |  |  | 0.99 | 1.02* | 1.01 |  | 1.00 | 1.01 | 1.00 |
|  |  |  |  |  |  |  |  |  |  |  |
|  |  |  |  |  |  |  |  |  |  |  |
| *Drinking Motives* | *N=942* |  |  | *N=939* | *N=727* | *N=526* |  | *N=908* | *N=333* | *N=276* |
| Enhancement | 1.03** |  |  | 1.05** | 0.95** | 0.98 |  | 1.05** | 0.98 | 0.98 |
| Social | 1.02* |  |  | 1.01 | 0.97** | 1.09** |  | 1.00 | 0.97 | 1.00 |
| Coping | 1.01 |  |  | 1.01 | 0.97* | 1.04 |  | 1.07** | 0.96* | 1.18** |
| Conformity | 1.01 |  |  | 0.98 | 1.05** | 0.94 |  | 1.00 | 1.02 | 0.86** |
|  |  |  |  |  |  |  |  |  |  |  |
|  |  |  |  |  |  |  |  |  |  |  |
| *Alcohol expectancies* | *N=980* |  |  | *N=961* | *N=741* | *N=537* |  | *N=931* | *N=340* | *N=281* |
| Risk & Aggression | 1.08** |  |  | 1.11** | 0.93 | 1.25* |  | 1.21** | 0.93 | 0.99 |
| Tension Reduction | 1.03 |  |  | 1.14** | 0.91* | 1.14 |  | 1.03 | 0.95 | 1.31 |
| Sociability | 1.10** |  |  | 1.07 | 1.02 | 1.22 |  | 1.05 | 1.01 | 1.13 |
| Sexuality | 0.99 |  |  | 0.98 | 0.98 | 0.78* |  | 1.05 | 0.96 | 0.76 |
| Liquid Courage | 1.02 |  |  | 1.01 | 1.04 | 0.71** |  | 0.92 | 0.98 | 0.95 |
| Cog & Beh Impair | 0.92** |  |  | 0.91** | 1.23** | 0.86 |  | 0.96 | 1.12* | 0.90 |
| Self-Perception | 0.95 |  |  | 0.99 | 0.96 | 0.96 |  | 1.15* | 0.94 | 1.23 |
|  |  |  |  |  |  |  |  |  |  |  |

* *p* < .05 ** *p* ≤ .01 *** *p* ≤ .001

HD = Heavy Drinking. AD = Alcohol Dependence. Externalizing = Antisocial Personality Disorder and Drug Dependence. Internalizing = Major Depression, Panic, Post-Traumatic Stress, and Generalized Anxiety Disorders. . Cog & Beh Impair = Cognitive and Behavioral Impairment.

Note: All models also included a control for the previous transition and demographic variables (age, income, and education level). Missing data for ages or other variables creates slight differences in numbers of cases counted towards events across models.
